# The Role of *β*-Catenin in Th1 Immune Response against Tuberculosis and Profiles of Expression in Patients with Pulmonary Tuberculosis

**DOI:** 10.1155/2021/6625855

**Published:** 2021-02-11

**Authors:** Kunlong Xiong, Jinxia Niu, Ruijuan Zheng, Zhonghua Liu, Yanzheng Song, Lin Wang, Changtai Zhu, Lin Fan

**Affiliations:** ^1^Shanghai Clinical Research Center for Tuberculosis, Shanghai Key Lab of Tuberculosis, Shanghai Pulmonary Hospital, Tongji University School of Medicine, Shanghai, China; ^2^Shanghai Public Health Clinical Center, Shanghai, China; ^3^Department of Laboratory Medicine, Shanghai Jiao Tong University Affiliated Sixth People's Hospital, Shanghai, China

## Abstract

*β*-Catenin is a key molecule of canonical Wnt/*β*-catenin pathway. Its roles and expression profiles in T cells of tuberculosis (TB) remain unclear. The aim of this study was to explore the role of *β*-catenin in CD4^+^ T cells and its expression characteristics in patients with pulmonary tuberculosis (PTB). In this study, CD4^+^ T cell-specific *β*-catenin conditional knockout mice (*β*-CAT-cKO mice) were aerosol infected with *Mycobacteria tuberculosis* (*Mtb*) H37R_V_ with wild-type mice as controls. Four weeks after infection, the mRNA expression of IFN-*γ*, TNF-*α*, and TCF-7 in the lungs of mice was measured. CD4, CD8, *β*-catenin, IFN-*γ*, and TNF-*α* in mononuclear cells from the lungs and spleens were measured by flow cytometry, and the pathological changes of lungs were also observed. Patients with PTB were enrolled, with blood samples collected and PBMCs isolated. The expressions of *β*-catenin, IFN-*γ*, TNF-*α*, and PD-1 in CD4^+^ and CD8^+^ T cells were measured by flow cytometry. Results showed a decreased frequency of and reduced IFN-*γ*/TNF-*α* mRNA expression and secretion by CD4^+^ T cells in the lungs of infected *β*-CAT-cKO mice compared with infected wild-type controls, and only slightly more inflammatory changes were observed in the lungs. *β*-catenin expressions in CD4^+^ and CD8^+^ T cells were significantly decreased in blood cells of patients with severe PTB compared with those in mild PTB. The stimulation of peripheral blood mononuclear cells (PBMCs) with lithium chloride (LiCl), a stimulant of *β*-catenin, resulted in the increase in CD4^+^ T cell frequency, as well as their secretion of IFN-*γ* and TNF-*α*. *β*-Catenin demonstrated a moderately positive correlation with PD-1 in CD4^+^ T cells. *β*-Catenin along with PD-1 and IFN-*γ* in CD4^+^ T cells had a high correlation with those in CD8^+^ T cells. In conclusion, *β*-catenin may be involved in the regulation of Th1 response and CD4^+^ T cell frequency in TB.

## 1. Introduction

Tuberculosis (TB) remains one of the main global causes of morbidity and mortality as an infectious disease. However, the immune response and its regulation mechanism in the host have not yet been cleared. *β*-Catenin is a key molecular of canonical Wnt/*β*-catenin pathway which is involved in the control of cell proliferation, differentiation, and differentiation in embryonic development [1]; CD4^+^ T cells play essential roles in controlling TB, and CD8^+^ T cells exhibit protection function against TB as well [2]. *β*-Catenin can regulate the T cell factor 1 (TCF1) in TCR signaling for differentiation of CD4^+^ T cells [3]. TCF1 or stabilized *β*-catenin can greatly stimulate the expression of the CD4 reporter gene by CD4 enhancer and promoter [4].

In the studies of *β*-catenin-related diseases, numerous published studies associate *β*-catenin, as a candidate antitumor therapeutic target, with the pathogenic process of malignant diseases by the regulation of cell differentiation [5–8]. Other studies indicated that Wnt/*β*-catenin signaling plays a role in the immune regulation of infectious diseases [1, 9]. Wnt/*β*-catenin canonical pathway could exhibit its regulation function in innate immunity against TB [1]. It is reported Wnt/*β*-catenin signaling could inhibit Bacillus Calmette-Guerin- (BCG-) infected macrophage autophagy and necrosis [10, 11]. Wnt/*β*-catenin is able to promote mitochondria-dependent apoptosis pathway in BCG-infected macrophage [10, 12].

*CTNNB1* gene has been shown to have gene polymorphisms of tuberculosis susceptibility in the Chinese Tibetan population [13]. Our previous studies [14] showed impaired IFN-*γ* expression and decreased CD4^+^ T cells in the blood of patients with severe cavitary PTB. A further study [15] indicated that *β*-catenin might be involved in the impaired IFN-*γ* expression, Th1 immune response of the host against TB, and mRNA of *β*-catenin, and TCF-7 was impaired in the CD14^−^ cells in the blood of patients with severe cavitary PTB.

In the present study, the role of *β*-catenin in the immunological regulation of CD4^+^ T cells was further explored. We constructed the CD4^+^ T cell-specific *Ctnnb1* conditional knockout mice and infected the mice with *Mtb* H37Rv through aerosol infection. Then, patients with active PTB were enrolled to determine the expression profiles of *β*-catenin and its related cytokines in human blood.

## 2. Materials and Methods

### 2.1. Patients

The patients hospitalized were recruited and satisfied the included criteria; the blood samples were collected from August 1, 2018, to March 31, 2019, at Shanghai Pulmonary Hospital, Tongji University. The included criteria were as follows: patients were confirmed as having active pulmonary tuberculosis (PTB) with *Mtb* culture positive or sputum acid-fast bacilli (AFB) smear positive with typical radiological changes consistent with TB manifestation or PCR amplification of *Mtb* positive. All patients included could be classified into severe lesion PTB and mild lesion PTB and had not been previously treated by anti-TB chemotherapy or been treated within one week. The method of classification was decided on the radiological manifestation and clinical characteristics previously described in our previous studies [14, 15]. Severe lesion PTB was defined as patients with a cavity of ≥3 cm in diameter or more than 3 cavities regardless of the diameter of the cavity; mild lesion PTB was defined as patients with no cavity of lesions in ≤2 lung fields. The study got the approval of the ethical committee of Shanghai Pulmonary Hospital; the approval number was fk17-022. All included patients signed the informed consent to participate in the study.

Patients who were in immunosuppressive status (including serum HIV-positive subjects) or were using immunosuppressive agents and accompanied by severe complications such as diabetes mellitus, malignant diseases, and liver diseases were excluded from this study.

### 2.2. Mice and *Mtb* Infection

All mice used in this study have C57BL/6 genetic background. *β*-CAT-cKO mice were generated by breeding mice bearing a LoxP-flanked gene *Ctnnb1* (*Ctnnb1^flox/flox^*) with *CD4-Cre* mice (mice expressing Cre recombinase under the control by CD4 promoter). Both *Ctnnb1^flox/flox^* and *CD4-Cre* mice were provided by Jackson Laboratory. To generate *CD4-Cre/Ctnnb1^flox/flox^* mice, homozygous *Ctnnb1^flox/flox^* mice were bred with *CD4-Cre* mice, and then, the offspring with genotype *CD4-Cre^+/-^/Ctnnb1^+/flox^* were intercrossed. The genotype was identified by PCR. Genomic DNA was prepared following standard tail clipping and DNA extraction procedures. PCR products were loaded in 2.5% agarose gel dyed with ethidium bromide for band visualization, and the primers used are listed in Table 1. All mice were bred in specific pathogen-free conditions at the Shanghai Model Organisms Center, Inc. Mice (6-8 weeks old) were divided randomly into cages upon arrival and infected by the nasal dropping of 5 × 10^4^ cfu*Mtb* (H37Rv) in the Biosafety Level-3 (BSL-3) Laboratory. The age-matched wild-type littermates were used as controls. All mice experiments were performed in accordance with the University Health Guide for the Care and Use of Laboratory Animals and were approved by the Ethics Committee of Shanghai Pulmonary Hospital.

### 2.3. Bacteria

*Mtb* H37Rv were grown in Middlebrook 7H9 broth (BectonDickinson, Cockeysville, MD) with 0.05% Tween-80 and 10% oleic acid-albumin-dextrose-catalase (OADC) (BectonDickinson, Cockeysville, MD).

### 2.4. Preparation of Single-Cell Suspension and Flow Cytometry

Mice were sacrificed by cervical dislocation at 28 days after infection. The lungs and spleens were collected and cut with scissors, then digested by pancreatin at 37°C with continuous agitation for 1 hour in a temperature-controlled water bath. After digestion, digested tissues were filtered through a sheet of mesh (70 *μ*m) screen into a 15 ml centrifuge tube for single-cell suspension; the tissues left were homogenized by grinding and also filtered into the 15 ml centrifuge tube. After washed by 1 ml RPMI-1640 (Gibco Invitrogen, Carlsbad, USA), the cells were resuspended with RPMI-1640 (supplemented with 10% calf serum, 100 U/l penicillin, and 100 U/l streptomycin) and diluted to 5 × 10^6^ cells/ml.

The isolated single-cell suspension was incubated at 37°C in a 5% CO_2_ incubator with 1 *μ*l/ml Brefeldin A (BFA, BD) for 5 hours. Then, cells were washed with RPMI-1640 and resuspended in flow cytometry staining buffer for surface marker staining. The cells were incubated with antimouse CD3-Pacific Blue (Clone 17A2, BioLegend) (1 : 200), CD4-BV510 (Clone RM4-5, BD Horizon™) (1 : 200), and CD69-BV605 (Clone H1.2F3, BioLegend) (1 : 100) antibodies for 30 minutes in the dark at 4°C. After surface marker staining, cells were permeabilized with fixation/permeabilization buffer (Transcription Factor Buffer Set, BD Pharmingen™) for 40 minutes at 4°C, then incubated with antimouse IFN-*γ*-BV711 (Clone XMG1.2, BD Horizon™) (1 : 100), *β*-catenin-Alexa 488 (Clone 14/Beta-Catenin, BD Transduction Laboratories™) (1 : 100) and TNF-*α*-PE-Cy7 (Clone MP6-XT22, BioLegend) (1 : 100) antibodies for 30 minutes in the dark at 4°C for intracellular cytokines staining; then, cells were washed by perm/wash buffer (included in Transcription Factor Buffer Set).

On account, *β*-catenin is a nuclear transcription factor, which expresses in both the cell cytoplasm and nucleus. Transcription Factor Buffer Set was used in this study for intracytoplasmic and intranuclear staining of *β*-catenin.

BD Canto II flow cytometer (BD Biosciences) was used to analyze the expression of surface markers and intracellular cytokines, and at least 10,000 events were collected. The results were analyzed using the FlowJo version 10.0.7 software (Tree Star, Ashland, OR, USA).

### 2.5. Histological Analysis

Following fixation with 4% phosphate-buffered formalin for 24 h, the lung tissues from *Mtb*-infected mice were embedded in paraffin wax and were cut into serial sections of 2-3 *μ*m thickness. The infiltration of immune cells into the lungs was detected by haematoxylin and eosin (H&E) staining.

### 2.6. RT-PCR Analysis

RNA from lungs and spleens of mice was extracted using TRIzol reagent (Invitrogen, Carlsbad, CA, USA). We performed cDNA synthesis using ReverTra Ace® qPCR RT kits (FSQ-101, Toyobo), and the relative mRNA expression of different genes was measured by quantitative real-time PCR with SYBR® Green Realtime PCR Master Mix kits (QPK-212, Toyobo) and calculated by comparison with the control gene *Actb* (encoding *β*-actin) using the 2^−△Ct^ method. The primers used are listed in Table 2.

### 2.7. Isolation of PBMCs

The included patients and healthy volunteers donated venous blood samples (10 ml) in heparin tubes; PBMCs (peripheral blood mononuclear cells) were isolated within six hours after drawn. PBMCs' isolation was performed by density gradient centrifugation using Ficoll-Paque Plus (GE healthcare, UK) following the manufacturer's instructions.

### 2.8. Human Cell Staining and Flow Cytometry

PBMCs were incubated at 37°C in a 5% CO_2_ incubator, and BFA (1 *μ*l/ml) was added to the cells 5 hours before staining. Antibodies were prepared, cells transferred to tubes then spun down at 350 g for 5 minutes at 4°C, and washed once in 500 *μ*l FACS buffer. Samples were resuspended in 100 *μ*l of diluted antibody against human cell-surface markers APC/H7-conjugated antihuman CD3 (Clone SK7, BD Pharmingen™) (1 : 200), BB515-conjugated antihuman CD4 (Clone PRA-T4, BD Horizon™) (1 : 200), and APC-conjugated antihuman CD279 (clone MIH4, BD Pharmingen™) (1 : 100); incubated for 30 minutes at 4°C; and washed twice in 500 *μ*l FACS buffer. Supernatants were removed, and cells were resuspended in 100 *μ*l of Cytofix/Cytoperm (BD Pharmingen™), then incubated for 40 minutes at 4°C. After washing, cells were suspended in diluted intracellular antibodies PerCP-Cy™5.5-conjugated antihuman IFN-*γ* (Clone B27, BD Pharmingen™) (1 : 100) and PE/Cy7-conjugated antihuman TNF-*α* (Clone MAb11, BioLegend) (1 : 100), incubated for 40 minutes at 4°C, then washed with 500 *μ*l perm/wash buffer. After cell staining, cells were resuspended in 100 *μ*l of 1% paraformaldehyde.

Data were collected using a BD Canto II flow cytometer (BD Biosciences), and at least 10,000 events were collected. The results were analyzed using FlowJo version 10.0.7 software (Tree Star, Ashland, OR, USA).

### 2.9. Activation of Wnt Pathway Tested by Flow Cytometry on PBMCs

PBMCs were incubated at 37°C in 5% CO_2_ incubator and stimulated with LiCl (cat NO. L9650, Sigma) at 20 mM for 24 hours before staining. BFA (1 *μ*l/ml) was added to the cells 5 hours before staining. Then, cells were collected and stained with APC/H7-conjugated antihuman CD3 (Clone SK7, BD Pharmingen™) (1 : 200), BB515-conjugated antihuman CD4 (Clone PRA-T4, BD Horizon™) (1 : 200), APC-conjugated antihuman CD279 (clone MIH4, BD Pharmingen™) (1 : 100), PerCP-Cy™5.5-conjugated antihuman IFN-*γ* (Clone B27, BD Pharmingen™) (1 : 100), PE/Cy7-conjugated antihuman TNF-*α* (Clone MAb11, BioLegend) (1 : 100), and PE-conjugated antihuman *β*-catenin (Clone 15B8, eBioscience™) (1 : 100).

### 2.10. Statistical Analysis

Statistical analysis was performed using the GraphPad Prism 8 software. Python 3.6 and module Seaborn Pandas were also used to analyze and plot the data. The data between the two groups were analyzed by two-tailed paired or unpaired Student *t*-test and expressed as mean ± standard deviation, and the chi-squared test was applied to examine some of the clinical characteristic parameters. The Pearson test was performed to determine the correlation between different expression profiles on CD4^+^ and CD8^+^ T cells, *p* < 0.05 for significant differences (^∗^*p* < 0.05; ^∗∗^*p* < 0.01; ^∗∗∗^*p* < 0.001; ^∗∗∗∗^*p* < 0.0001).

## 3. Results

### 3.1. Patient Population

Thirty-nine patients with PTB were recruited into the present study. Of them, 17 cases had severe PTB while 22 cases had mild PTB. All recruited subjects had evenly distributed ages and sexes and no severe complications with an average age of 34.8 ± 19.2 years old; males occupied 52.9% in severe PTB and 59.1% in mild PTB. Detailed characteristics of severe and mild lesion PTB are shown in Table 3.

### 3.2. Frequency of *Mtb* Antigen-Specific CD4^+^ T Cells Was Decreased in Infected *Ctnnb1^−/−^* Mice

**Figure 1:**
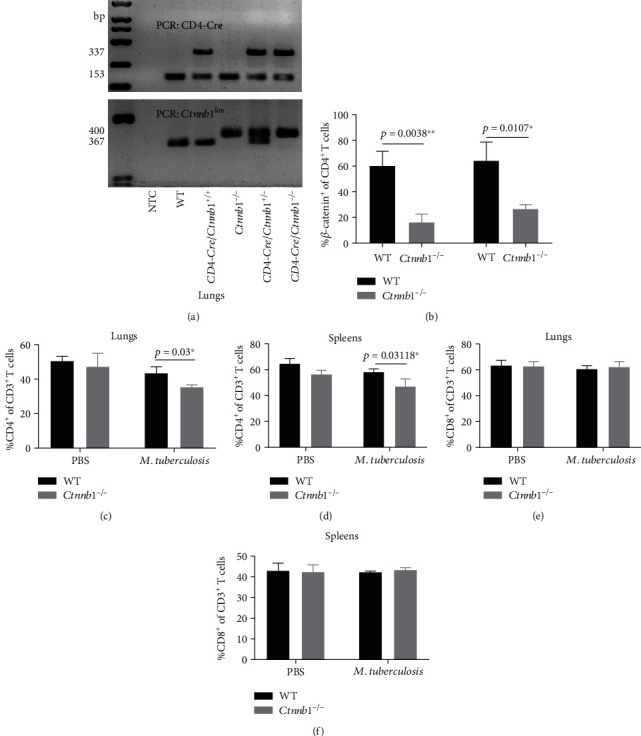
The genotypes of all mice identified by PCR and T cells frequency in both groups of mice by flow cytometry. (a) Mice's genotypes were identified by PCR, and results were showed by 2.5% agarose gel electrophoresis. (b) The frequency of *β*-catenin^+^ cells of CD4^+^ T cells in lungs and spleens of *Ctnnb1^−/−^* mice (*n* = 5) and wild-type mice (*n* = 5); CD3^+^CD4^+^ T cells were shown in the lungs (c) and spleens (d) of *Ctnnb1*^−/−^ (*n* = 5) and wild-type mice (*n* = 5) infected with or without *M. tuberculosis*; CD3^+^CD8^+^ T cells were shown in the lungs (e) and spleen (f) of *Ctnnb1*^−/−^ (*n* = 5) and wild type mice (*n* = 5) infected with or without *M. tuberculosis*. The experiment had three independent rounds, and results were analyzed by the Student *t* test. NTC, no template control. PBS, phosphate-buffered saline.

The genotypes of all mice were identified by PCR (Figure 1(a)), and we use the genotype *CD4-Cre*/*Ctnnb1^−/−^* mice as *Ctnnb1^−/−^* mice for further research. As shown in Figure 1(b), the frequency of *β*-catenin^+^ cells in both lung and spleen CD4^+^ T cells was significantly decreased in *Ctnnb1^−/−^* mice compared with that in wild-type mice (*p* = 0.0038 and 0.0107, respectively). To confirm the changes of CD4^+^ T cells after *Ctnnb1^−/−^* mice were infected with *Mtb* H37Rv, we measured the frequency of CD4^+^ T cells in the cells from lungs and spleens of mice by flow cytometry. Results showed that after *Mtb* H37Rv infection, a significantly decreased frequency of CD4^+^ T cells in the lungs and spleens from *Ctnnb1*^−/−^ mice was observed compared with wild-type mice (*p* = 0.03 and 0.03118, respectively) (Figures 1(c) and 1(d)). However, the CD8^+^ T cells from the lungs and spleens were similar between *Ctnnb1*^−/−^ and wild-type mice (Figures 1(e) and 1(f)).

### 3.3. *Mtb* Antigen-Specific Production of IFN-*γ* and TNF-*α* in CD4^+^ T Cells Decreased in the Lungs of *Ctnnb1*^−/−^ Mice

**Figure 2:**
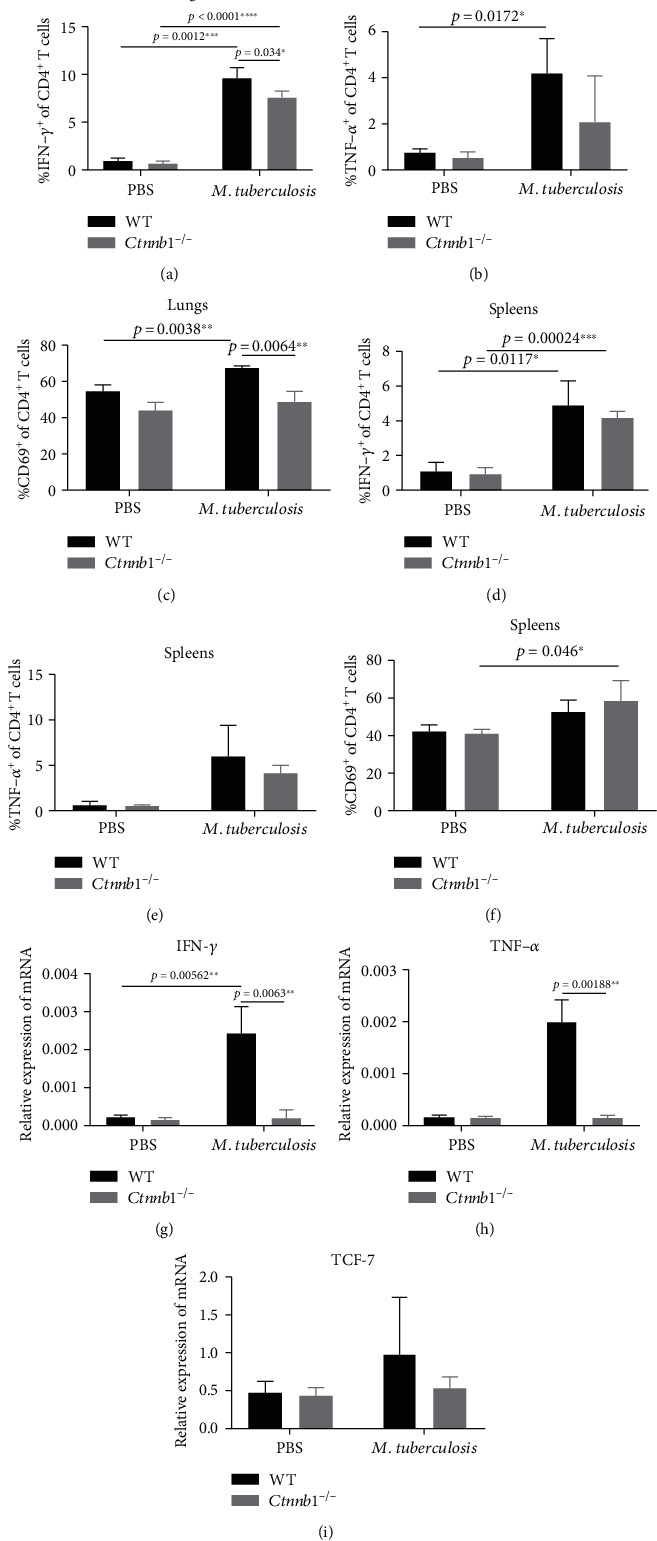
The expression of IFN-*γ* and TNF-*α* in CD3^+^ and CD4^+^ T cells. (a, b) IFN-*γ*^+^CD4^+^ T cell in the lungs and spleens of *Ctnnb1^−/−^* mice (*n* = 5) and wild-type mice (*n* = 5) infected with or without *M. tuberculosis*. (c, d) TNF-*α*^+^CD4^+^ T cell in lungs and spleens of *Ctnnb1^−/−^* mice (*n* = 5) and wild-type mice (*n* = 5) infected with or without *M. tuberculosis*. (e, f) CD69^+^CD3^+^CD4^+^ T cells in the lungs and spleens from *Ctnnb1^−/−^* mice (*n* = 5) and wild-type mice (*n* = 5) infected with or without *M. tuberculosis*. (g, i) mRNA of IFN-*γ*, TNF-*α*, and TCF-7 in the lungs of *Ctnnb1^−/−^* mice (*n* = 5) and wild-type mice (*n* = 5) infected with or without *M. tuberculosis*. The experiment had three independent rounds, and results were analyzed by the unpaired Student *t*-test. PBS, phosphate-buffered saline.

To further verify the changes of Th1 response since *β*-catenin in CD4^+^ T cells was knocked out in *Ctnnb1*^−/−^ mice, we tested IFN-*γ* and TNF-*α* secreted by CD4^+^ T cells in both lungs and spleens. Results showed that *Mtb* antigen-specific IFN-*γ* production and CD69^+^ expression CD4^+^ T cells in lungs were significantly decreased in *Ctnnb1*^−/−^ mice compared with wild-type mice (*p* = 0.034 and 0.0064, respectively) (Figures 2(a) and 2(c)), indicating *β*-catenin is able to promote the Th1 cytokine production and activate CD4^+^ T cells, although the difference in frequency of TNF-*α*^+^CD3^+^CD4^+^ T cells had no statistical significance between the two groups in the lungs (Figure 2(b)). Meanwhile, *Mtb* antigen-specific IFN-*γ* and TNF-*α* production by CD4^+^ T cells and the frequency of CD69^+^CD4^+^ T cells in the spleens was kept similar between the infected *Ctnnb1*^−/−^ and wild-type mice (Figures 2(d)–2(f)). IFN-*γ* and TNF-*α* mRNA in the lungs of infected *Ctnnb1*^−/−^ mice were significantly decreased compared with those in infected wild-type mice (*p* = 0.0063 and 0.00188, respectively), while no difference was found in TCF-7 mRNA between two groups of mice (Figures 2(g)–2(i)).

In addition, the expression of IFN-*γ*, TNF-*α*, CD69, and IFN-*γ* mRNA in the lung CD4^+^ T cells and IFN-*γ* in spleen CD4^+^ T cells significantly elevated after infection in WT mice (*p* = 0.00012, 0.0172, 0.0038, 0.00562, and 0.0117, respectively) (Figure 2).

### 3.4. The Lungs of *Ctnnb1*^−/−^ Mice Exhibited Slightly More Pathological Inflammation and Similar Bacterial Load Compared with Wild-Type Mice

**Figure 3:**
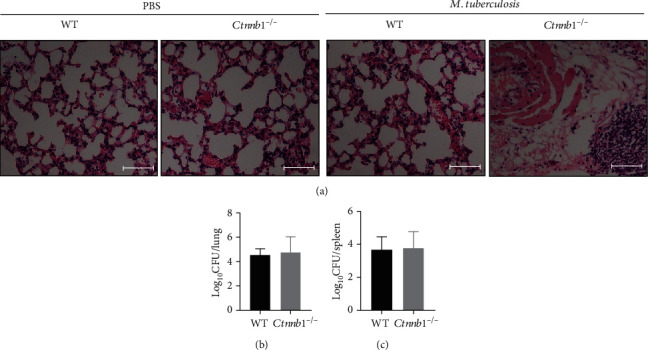
Histopathology and bacterial load of lungs (and spleens) of mice with *Mtb* infection in vivo. (a) Representative histopathology in lung sections stained with haematoxylin and eosin (H&E) of wild-type mice and *Ctnnb1^−/−^* mice infected with or without *M. tuberculosis* for 4 weeks (bar, 100 *μ*m; original magnification ×200). (b) Bacterial load (cfu) in the lungs of both *Ctnnb1*^−/−^ (*n* = 5) and wild-type mice (*n* = 5) infected with *M. tuberculosis* for 4 weeks. (c) Bacterial load (cfu) in the spleens of both *Ctnnb1*^−/−^ (*n* = 5) and wild-type mice (*n* = 5) infected with *M. tuberculosis* for 4 weeks. The experiment had three independent rounds, and results were analyzed by the unpaired Student *t*-test. PBS, phosphate-buffered saline.

The lung tissue was collected, with the pathological changes observed in infected *Ctnnb1*^−/−^ mice. We found that the lungs in *Ctnnb1*^−/−^ mice exhibited more profound inflammatory changes compared with those in wild-type mice infected (Figure 3(a)). However, we found no differences in bacterial load in lungs and spleens between *Ctnnb1*^−/−^ and wild-type mice (*p* = 0.7098 and 0.899, respectively) (Figures 3(b) and 3(c)).

### 3.5. Expression of *β*-Catenin in CD4^+^ T Cells Was Decreased in PBMCs of Patients with Severe PTB

**Figure 4:**
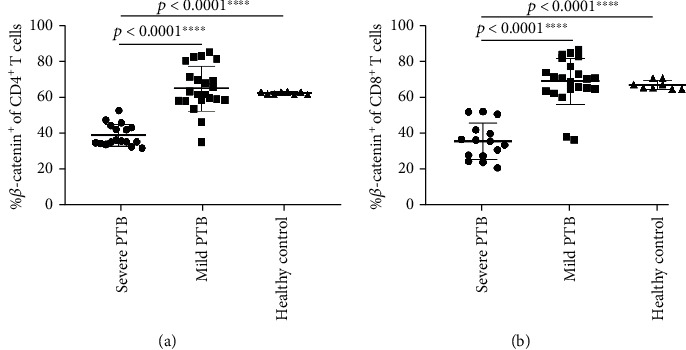
Expression of *β*-catenin on CD4^+^ and CD8^+^ T cells. PBMCs from patients with severe PTB (*n* = 17), mild PTB (*n* = 22), and health controls (*n* = 8), then stained the PBMCs by flow cytometry. (a) *β*-catenin expression in CD4^+^ T cells. (b) *β*-catenin expression in CD8^+^ T cells. Results were analyzed by the unpaired Student *t*-test.

To determine the expression characteristics of *β*-catenin in T cells of PTB patients, we tested the *β*-catenin in PBMCs of PTB patients by flow cytometry. As the nuclear molecules can be stained with flow cytometry antibodies by using Transcription Factor Buffer Set (BD Pharmingen™), we could observe a very high frequency of *β*-catenin^+^ cells in most samples. The results showed that *β*-catenin in CD4^+^ T cells was decreased significantly in patients with severe PTB than that in patients with mild PTB (*p* < 0.0001), *β*-catenin in CD4^+^ T cells of patients with severe PTB was accordingly significantly lower than that in healthy donors (*p* < 0.0001), while *β*-catenin expression was similar between patients with mild PTB and healthy donors (*p* = 0.5771) (Figure 4(a)).

### 3.6. Expression of *β*-Catenin in CD8^+^ T Cells in PBMCs of Patients with Severe PTB and Its Lower Frequency Compared with That in Patients with Mild PTB

To compare the expression of *β*-catenin in CD8^+^ T cells of PTB patients, we tested *β*-catenin expression in CD8^+^ T cells of PTB patients by flow cytometry. The results showed that *β*-catenin in CD8^+^ T cells was decreased significantly in patients with severe PTB than that in patients with mild PTB and healthy donors as well (*p* < 0.0001, both), while *β*-catenin expression was similar in between patients with mild PTB and healthy donors (*p* = 0.6897) (Figure 4(b)).

### 3.7. *β*-Catenin Can Promote Th1 Cytokine Secretion and Increase the Frequency of CD4^+^ T Cells, rather than the PD-1 Expression, in Patients with PTB

**Figure 5:**
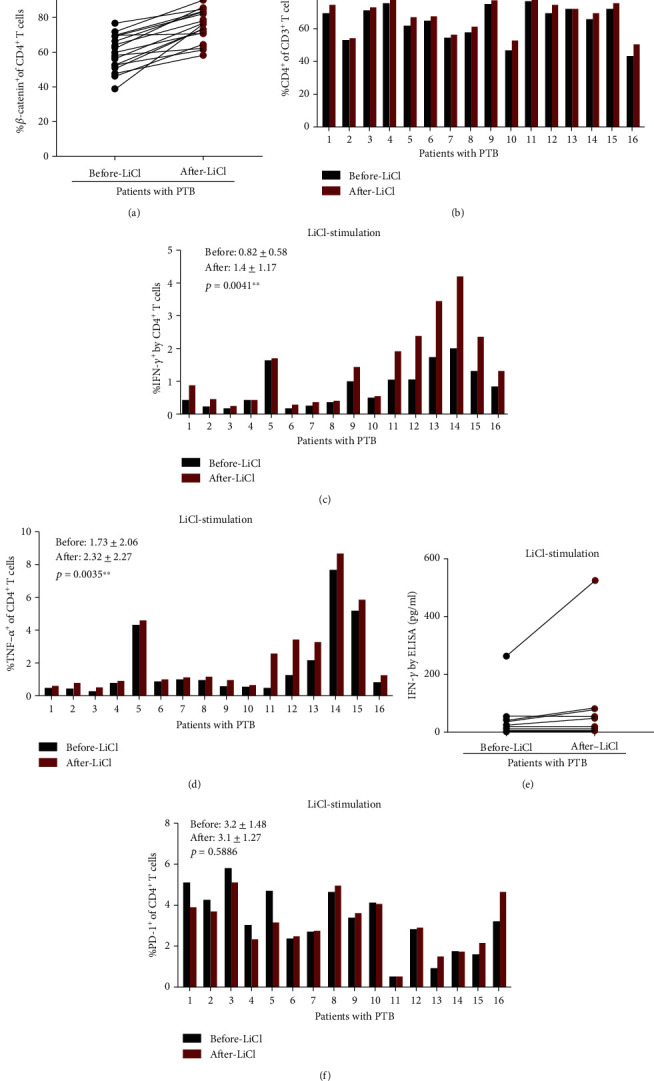
PBMCs cultured with LiCl to stimulate the production of *β*-catenin. (a) Significant increase in the *β*-catenin expression on CD4^+^ T cells after LiCl stimulation, *n* = 16. (b) Significant increase in the CD4^+^ frequency after LiCl stimulation, *n* = 16. (c) Significant increase in the IFN-*γ* expression on CD4^+^ T cells after LiCl stimulation, *n* = 16. (d) Significant increase in the TNF-*α* expression on CD4^+^ T cells after LiCl stimulation, *n* = 16. (e) The ELISA result showed similar IFN-*γ* expression before after LiCl stimulation, *n* = 10. (f) No changes of PD-1 expression on CD4^+^ T cells after LiCl stimulation, *n* = 16. All results were analyzed by the paired Student *t*-test.

LiCl, a stimulant of *β*-catenin, was added into the cultures of PBMCs to increase the *β*-catenin expression. The results showed that the percentage of *β*-catenin expressing in CD4^+^ T cells was increased significantly (*p* < 0.0001), and CD4^+^ T cells were also significantly increased under stimulation of LiCl (*p* < 0.0001). Meanwhile, Th1 cytokines produced by CD4^+^ T cells, including IFN-*γ* and TNF-*α*, were significantly increased after the simulation of LiCl (*p* = 0.0041 and 0.0035, respectively) (Figures 5(a)–5(d)), while the result of ELISA showed similar IFN-*γ* secretion in PTB patients before and after stimulation of LiCl (before -LiCl group vs. after -LiCl group = 82.88 ± 151.17 vs. 45.25 ± 74.98, *p* = 0.177) (Figure 5(e)). In addition, PD-1 expression in CD4^+^ T cells was not obviously increased or decreased under the stimulation of LiCl (*p* = 0.5886) (Figure 5(f)).

### 3.8. *β*-Catenin Showed a Weak Positive Correlation with PD-1 Expression and Had No Correlation with IFN-*γ* on Both CD4^+^ and CD8^+^ T Cells

**Figure 6:**
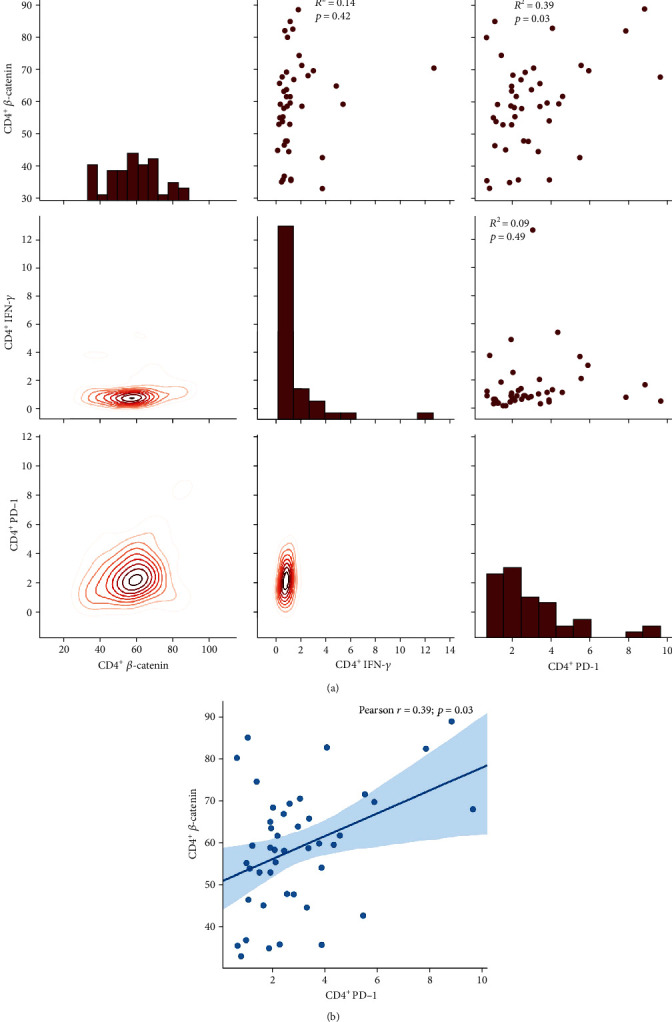
(a) The correlation of *β*-catenin, PD-1, and IFN-*γ* on CD4 T cells. (b) The correlation of *β*-catenin and PD-1 on CD4 T cells (*n* = 47).

**Figure 7:**
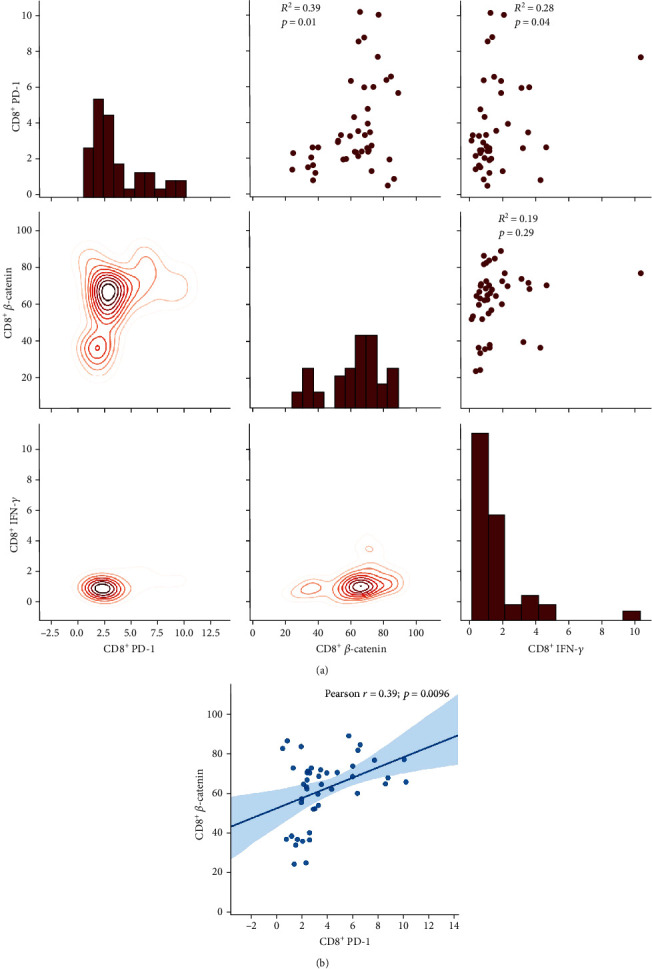
(a) The correlation of *β*-catenin, PD-1, and IFN-*γ* on CD8 T cells. (b) The correlation of *β*-catenin and PD-1 on CD8 T cells (*n* = 47).

We tested the expression of *β*-catenin, IFN-*γ*, and PD-1 in CD4^+^ T cells from PBMCs of the same group of PTB patients through flow cytometry. The data showed that *β*-catenin had moderately positive correlation with PD-1 (*R*^2^ = 0.39; *p* = 0.03) (*R*^2^: Pearson *r* score) (Figures 6(a) and 6(b)). We also tested the expression of *β*-catenin, IFN-*γ*, and PD-1 in CD8^+^ T cells from PBMCs of the same group of PTB patients through flow cytometry. The data showed that the production of *β*-catenin and PD-1 were also positively moderately correlated (*R*^2^ = 0.39; *p* = 0.0096), and the correlation of CD8^+^PD-1^+^ and CD8^+^ IFN-*γ*^+^ cells was weak to moderate (*R*^2^ = 0.28; *p* = 0.04) (Figures 7(a) and 7(b)). The molecular mechanisms underlying the positive correlation between *β*-catenin and PD-1 remain to be determined.

### 3.9. *β*-Catenin, PD-1, and IFN-*γ* in CD4^+^ T Cells Are Highly Correlated with Their Respective Expression in CD8^+^ T Cells

**Figure 8:**
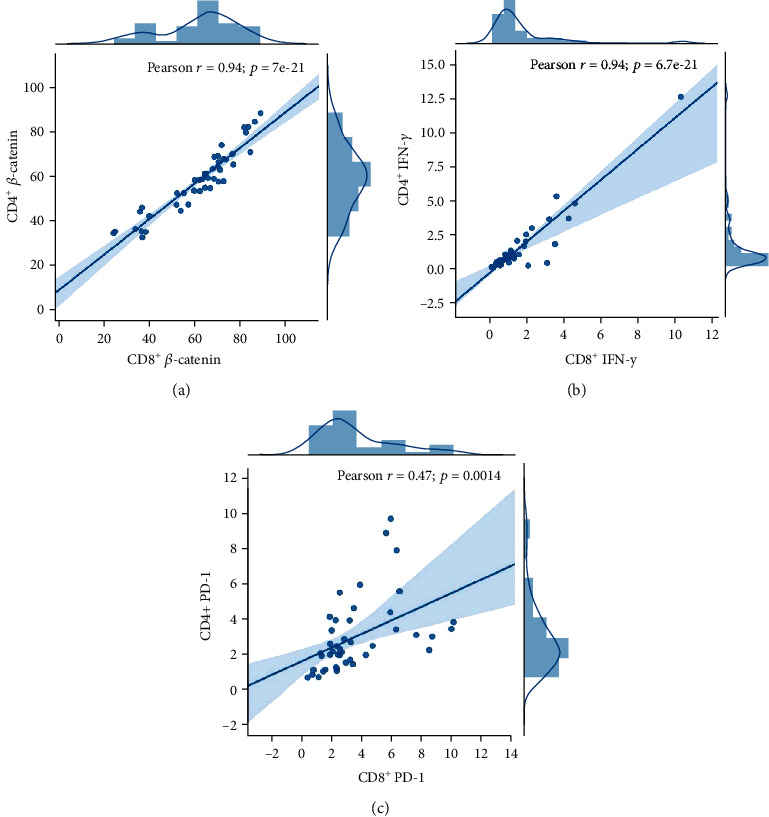
(a) The correlation of *β*-catenin on CD4 T cells and *β*-catenin on CD8 T cells. (b) The correlation of IFN-*γ* on CD4 T cells and IFN-*γ* on CD8 T cells. (c) The correlation of PD-1 on CD4 T cells and PD-1 on CD8 T cells (*n* = 47).

After the analysis of the expression level of *β*-catenin and IFN-*γ* in CD4^+^ and CD8^+^ T cells, we found *β*-catenin in CD4^+^ T cells was highly correlated with that in CD8^+^ T cells, with *R*^2^ equal to 0.94 (*p* = 7e − 21). IFN-*γ* in CD4^+^ cell was also highly associated with its expression in CD8^+^ T cell, with *R*^2^ equal to 0.94 (*p* = 6.7e − 21), PD-1 in CD4^+^ T cells moderately associated with its expression in CD8^+^ T cells, and *R*^2^ was 0.47 (*p* = 0.0014). The data are shown in Figures 8(a)–8(c).

## 4. Discussion

*β*-Catenin has been thought to be involved in the regulation of cell fate, such as proliferation, differentiation, and apoptosis or T cell development [16, 17]. In most published studies, *β*-catenin has a close association with the pathogenesis of malignant diseases and is a therapeutic target for cancer in view of its function to promote tumor cell proliferation [18]. *β*-Catenin is a core molecule in the canonical Wnt/*β*-catenin pathway, through the ubiquitin-proteasome system (UPS); it is kept at a low level in the absence of Wnt ligands. Upon Wnt activation, *β*-catenin accumulates in the cytoplasm, then enters into the nucleus and binds to some coregulators to promote the transcription of some genes, such as *CyclinD* (*cycD*), *c-myc*, and *c-Jun*, which, in turn, regulate the cell fate. Thus, the protein level of *β*-catenin in the whole cell can be associated with the activation of the canonical Wnt/*β*-catenin pathway. Our previous study showed that *β*-catenin mRNA in CD14^−^ cells was impaired in severe PTB patients, and stimulation of *β*-catenin could enhance the activation of CD4^+^ T cells in the blood of PTB [15]. In the present study, the frequency of CD4^+^ T cells in conditional *Ctnnb1*^−/−^ infected mice was decreased, indicating a role of *β*-catenin in the activation and proliferation of CD4^+^ T cells of hosts infected with TB.

Results from Kared et al. [19] indicate that the Wnt/*β*-catenin axis may represent a key pathway in reversing T cell defects and was associated with the heterogeneity of CD4^+^ T cells in humans. Our studies also showed that Th1 response was impaired in *Ctnnb1*^−/−^ infected mice, implying that the Wnt/*β*-catenin pathway might play a role in the process of regulating CD4^+^ T cell activation, differentiation, and Th1 response in host with TB. Meanwhile, the study showed the slightly more obvious inflammation cells gathering in *Ctnnb1*^−/−^ mice compared to wild-type mice, while both groups demonstrated similar bacteria loads in both lungs and spleens, implying that *β*-catenin might participate in the regulation of functional effects of Th1 and proliferation of CD4^+^ T cells, leading to immunological damage. We did not find the change in bacterial load in *Ctnnb1*^−/−^ mice, which was possibly stemmed from multiple other factors involved in the bacteria growth.

To further confirm the conclusions in mice experiments, we recruited a group of PTB patients, who were divided into mild and severe groups. Then, we obtained blood samples, isolated PBMCs, and tested *β*-catenin expression in CD4^+^ and CD8^+^ T cells by flow cytometry. We also stained the cells with anti-IFN-*γ*, TNF-*α*, and PD-1 antibodies. We found that *β*-catenin expression in both CD4^+^ and CD8^+^ T cells was significantly impaired in severe compared to mild subjects. Unlike our previous study, the *β*-catenin expression also underwent remarkable changes in CD8^+^ T cells in PTB patients in a similar way as in CD4^+^ T cells. *β*-Catenin had been studied in the ovarian tumors, and results showed that the blocking Wnt/*β*-catenin pathway in CD8^+^ T cells at least partly explains the retardation of tumor growth and improvement of patient survival [20]. In T cell inflammation of tumor microenvironment, such as in colorectal cancer, Wnt/*β*-catenin pathway plays an essential role in CD8^+^ T cells activation [21], since intrinsic *β*-catenin can suppress the CD8^+^ T cells infiltration, resulting in the mediation of colorectal cancer resistance to chemotherapy [22]. The present TB study showed that *β*-catenin in both CD4^+^ and CD8^+^ T cells had a close association with the regulation of TB progress. Increased *β*-catenin can enhance the expression of Th1 responses in the CD4^+^ T population, which is consistent with our previous study [15]. Interestingly, the frequency of IFN-*γ* in CD4^+^ T cells was significantly increased in PBMCs of PTB patients after LiCl stimulation compared with before LiCl stimulation (Figure 5(c)), while IFN-*γ* measurements by ELISA of PTB patients' PBMCs did not show differences between before and after LiCl stimulation (Figure 5(e)), indicating that increased *β*-catenin did not enhance the whole IFN-*γ* secretion but IFN-*γ* secretion from some kinds of cells such as CD4^+^ T cells in the PBMCs of PTB patients.

As *β*-catenin was impaired in severe PTB, in whom the frequency of CD4^+^ T cells decreased [14], we wondered whether decreased CD4^+^ T cells and Th1 secretion was associated with changed PD-1 expression or impaired Th1 expression was due to PD-1 involvement. The results indicated that the variation in *β*-catenin could stimulate the CD4^+^ T cells and Th1 cytokines expression, though PD-1 expression on CD4^+^ T cells was not influenced. But the level of *β*-catenin showed a moderate positive correlation with PD-1 rather than Th1 cytokine production, implying the role of *β*-catenin had partly associated with PD-1 but had no direct or crucial association. In view of the role of *β*-catenin showed in a tumor study, in which *β*-catenin promote immune escape and impair T cells activity, resulting in resistance to anti-PD-1 therapy in hepatocellular carcinoma [23], *β*-catenin has been applied in the anticancer strategy of therapy in combination with PD-1 immunotherapy [24]. PD-1 has been thought to be an exhaustion marker on T cell surface [25, 26], suggesting decreased CD4^+^ T cells in severe PTB are associated with impaired *β*-catenin and T cell exhaustion.

The other interesting finding in the present study was that *β*-catenin, PD-1, and IFN-*γ* in CD4^+^ T cells had strong correlations with their respective expression in CD8^+^ T cells. In our earlier research, we observed a variation in the number of CD4^+^ T cells and their cytokine production, but not in CD8^+^ T cells between severe and mild PTB subjects, but we confirmed the decrease in the number of CD4^+^ T cells and impaired Th1 cytokine production in *Ctnnb1* conditional knocked mice as expected. However, in human studies, a similar changing trend of CD8^+^ T cells was found, indicating that *β*-catenin could participate in the regulation of cell proliferation and activation of both CD4^+^ and CD8^+^ T cells. In conclusion, *β*-catenin can promote CD4^+^ and CD8^+^ T cell activation and play a role in immune protection in TB infection.

In summary, this study focused on the role and expression of *β*-catenin in TB in both mouse experiments and human studies. The results showed *β*-catenin elevated T cell number and Th1 cytokine secretion by CD4^+^ T cells. *β*-Catenin may be involved in the pathogenesis of TB diseases by impairing CD4^+^ and CD8^+^ T cells, which is, at least, attributing to PD-1-induced exhaustion.

## Figures and Tables

**Table 1:** Primers used for identification.

Gene	Sequence 5′ → 3′	Reaction
*CD4-Cre*	GTTCTTTGTATATATTGAATGTTAGCC	Common
	TATGCTCTAGGACAAGAATTGACA	Wild-type reverse
	CTTTGCAGAGGGCTAACAGC	Mutant reverse
*Ctnnb1*	AAGGTAGAGTGATGAAAGTTGTT	Forward
	CCTGTTAGCCCTCATGGTGT	Reverse

**Table 2:** Primers used for qPCR.

Gene	Forward primer(5′→3′)	Reverse primer(5′→3′)
*Tcf-7*	AAGAAGAAGAGGCGGTCAAGG	CACTGTCATCGGAAGGAACG
*Ifn-γ*	TCAAGTGGCATAGATGTGGAAGA	TGCTGATGGCCTGATTGTCT
*Tnf-α*	ACTGGCAGAAGAGGCACTCC	GCCACAAGCAGGAATGAGAA
*Actb*	CCCAGTCCTTCACGCAAGAG	CATCTAGCGTCTCAGGGAACA

**Table 3:** Characteristics of patients with PTB.

Characteristics	Severe PTB (17)	Mild PTB (22)	*p* value
Age	30.7 ± 12.5	38.8 ± 13.3	0.62
Sex (male)	9 (52.9%)	13 (59.1%)	0.701
HIV positive	0	0	—
Accompanied by DM	0	0	—
Taking immune-suppressive agents	0	0	—
Malignant diseases	0	0	—
Smoking	6 (35.3%)	7 (31.8%)	0.82
Alcohol	2 (11.7%)	3 (13.6%)	0.86
Concurrent EPTB	3 (17.6%)	5 (22.7%)	0.697

PTB: pulmonary tuberculosis; EPTB: extrapulmonary tuberculosis; DM: diabetes mellitus.

## Data Availability

The data used to support the findings of this study are available from the corresponding author upon request.
